# Silver Chloride Precipitation-limiting Factor for Accurate Silver Determination in Ag-accumulating Mushrooms After Nitric Acid Digestion

**DOI:** 10.1007/s12011-025-04605-1

**Published:** 2025-04-14

**Authors:** Iva Synková, Jan Borovička

**Affiliations:** 1 Institute of Geochemistry, Mineralogy and Mineral Resources, Faculty of Science, Charles University in Prague, Albertov 6, 12843 Prague 2, Czech Republic; 2 Czech Academy of Sciences, Nuclear Physics Institute, Hlavní 130, 25068 Husinec-Řež, Czech Republic; 3 Czech Academy of Sciences, Institute of Geology, Rozvojová 269, 16500 Prague 6, Czech Republic

**Keywords:** Fungi, Hyperaccumulation, INAA, HR-ICP-MS, ICP-OES, Microwave digestion, Standard reference materials

## Abstract

Wild-growing mushrooms (macromycetes, macrofungi) are capable of accumulating high amounts of trace elements, including noble metals. In a series of analytical experiments, this study focused on the problem of correct determination of Ag mass fractions in the biomass of Ag-accumulating mushrooms, Ag-hyperaccumulators in particular. A unique experimental setup enabled to compare Ag determination in selected mushroom samples first by the non-destructive neutron activation analysis (INAA) utilizing ^110^Ag isotope, second by the high-resolution inductively coupled plasma mass spectrometry (HR-ICP-MS) after HNO_3_ hotplate and microwave digestions. Furthermore, Ag-spiking experiments were conducted with three mushroom species containing 860, 6200, and 21,000 mg Cl kg^−1^ in dry mass and Ag was determined by HR-ICP-MS after HNO_3_ hotplate digestion of the samples. Finally, ten replicates of Ag-hyperaccumulating *Amanita strobiliformis* were analyzed by two variants of INAA utilizing ^108^Ag in the first step and ^110 m^Ag isotope in the second step. In addition, four additional digestion procedures followed by HR-ICP-MS and inductively coupled plasma optical emission spectroscopy (ICP-OES) measurements of Ag were performed on this *A. strobiliformis* sample. Analytical quality was controlled by using NIST Standard Reference Materials 1566b (Oyster Tissue) and 2781 (Domestic Sludge). The results of all experiments revealed that correct determination of Ag in mushrooms is not always possible using standard digestion procedures because of precipitation of insoluble AgCl which was documented by scanning electron microscopy and confirmed by the X-ray diffraction analysis.

**Graphical Abstract:**

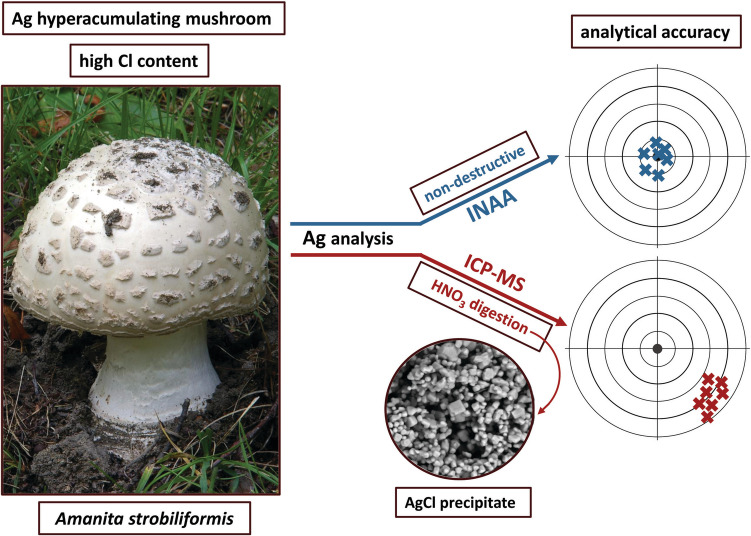

**Supplementary Information:**

The online version contains supplementary material available at 10.1007/s12011-025-04605-1.

## Introduction

Mushrooms (macrofungi, macromycetes) play a considerable environmental role in biogeochemical cycling of many chemical elements, including metals [1]. Since 1970 s, many studies have been published on trace element accumulation in mushroom fruit bodies from both clean and polluted environments. The early studies in this field used chromatographic and photometric methods [2–4], instrumental neutron activation analysis (INAA) [5, 6], or atomic absorption spectroscopy (AAS) [7–9]. Since the 1990 s, these methods were gradually replaced by ICP-based techniques: inductively coupled plasma mass spectrometry (ICP-MS) [10, 11] and inductively coupled plasma atomic/optical emission spectroscopy (ICP-AES, ICP-OES) [12–14].

Very high mass fractions of various toxic metals like Cd or Hg [15, 16], metalloids like V and As [17, 18], and noble metals like Au and Ag [6, 19–21] in mushrooms have attracted investigators from various research fields. Among all, mushroom hyperaccumulators occupy a special position and have been inspected for element uptake [22–24], intracellular sequestration [25–27], and speciation [28, 29].

**Fig. 1:**
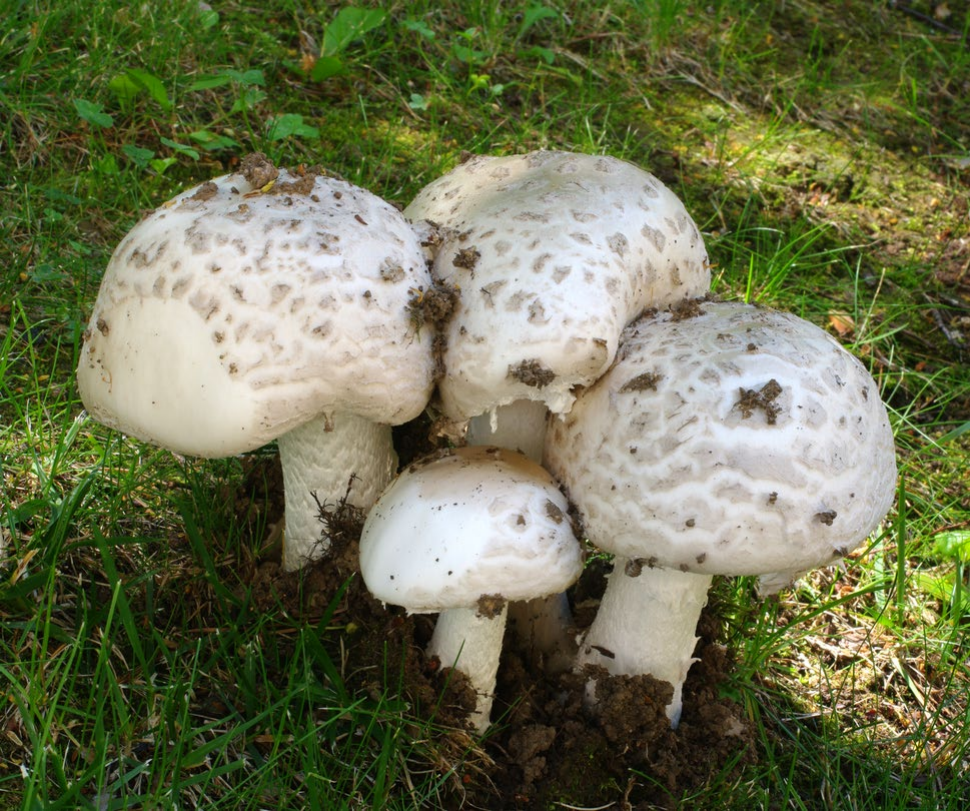
*Amanita strobiliformis* in its natural habitat, the most efficient known Ag-hyperaccumulating mushroom species. Photo by Jan Borovička

Three European *Amanita* species (*Agaricales*, *Amanitaceae*) have been found to hyperaccumulate Ag [30]. Among them, *Amanita strobiliformis* (Fig. 1) commonly accumulates hundreds of mg Ag kg^−1^ in dry mass (DM) in its fruit bodies, with the highest levels exceeding even 1200 mg kg^−1^ at sites with the background Ag content in soils. A field study using the soil sequential extraction procedure and Pb isotopic fingerprints revealed that the mycelium of *A. strobiliformis* for uptake likely attacks the reducible metal fraction of the colonized soil [31] and liberated Ag is translocated to fruit bodies. The cellular uptake of Ag can be facilitated by transporters from the copper transporter family, which in this species recognize and transport Ag in addition to the essential Cu [32]. Fruit body Ag remains stored in cells as monovalent ion tightly complexed with specialized metallothionein peptides in the cytoplasm [33, 34]. The biological or ecological significance of the Ag hyperaccumulation trait is unknown; however, it was demonstrated that the Ag liberated from decaying Ag-rich mushrooms significantly alters the composition of bacterial community in the underlying soil [35].

When we attempted to follow-up on the INAA-based study by Borovička et al. [30] on Ag hyperaccumulation in mushrooms using ICP-MS as the analytical method, we achieved unexpectedly low Ag mass fractions in *A. strobiliformis* samples. This striking analytical discrepancy was even more suspicious because both the INAA and ICP-MS data were supported by satisfactory results for Ag in the co-analyzed standard reference materials (SRMs).

That led us to a hypothesis that the problem could lie in the destructive nature of the ICP-MS analysis. Mushroom fruit-bodies may contain very high levels Cl, commonly reaching thousands of mg kg^−1^ in DM [36, 37]. This would suggest that insoluble AgCl is possibly precipitated during the biomass mineralization, which ultimately leads to incorrect determination of Ag mass fractions. An insight into our data has revealed that *A. strobiliformis* contains 1–2 wt% Cl in DM. To compare destructive and non-destructive analysis, we therefore designed an experiment in which selected mushroom samples were analyzed for Ag first by INAA, then by high-resolution inductively coupled plasma mass spectrometry (HR-ICP-MS). Furthermore, we conducted Ag-spiking experiments with mushroom biomass of different Cl content. A comparative analysis of Ag in *A. strobiliformis* was done using several INAA and ICP-based techniques. Finally, we unambiguously proved that AgCl is precipitated during nitric acid digestion of *A. strobiliformis* biomass by using scanning electron microscopy (SEM) and X-ray diffraction technique (XRD).

## Materials and Methods

**Fig. 2:**
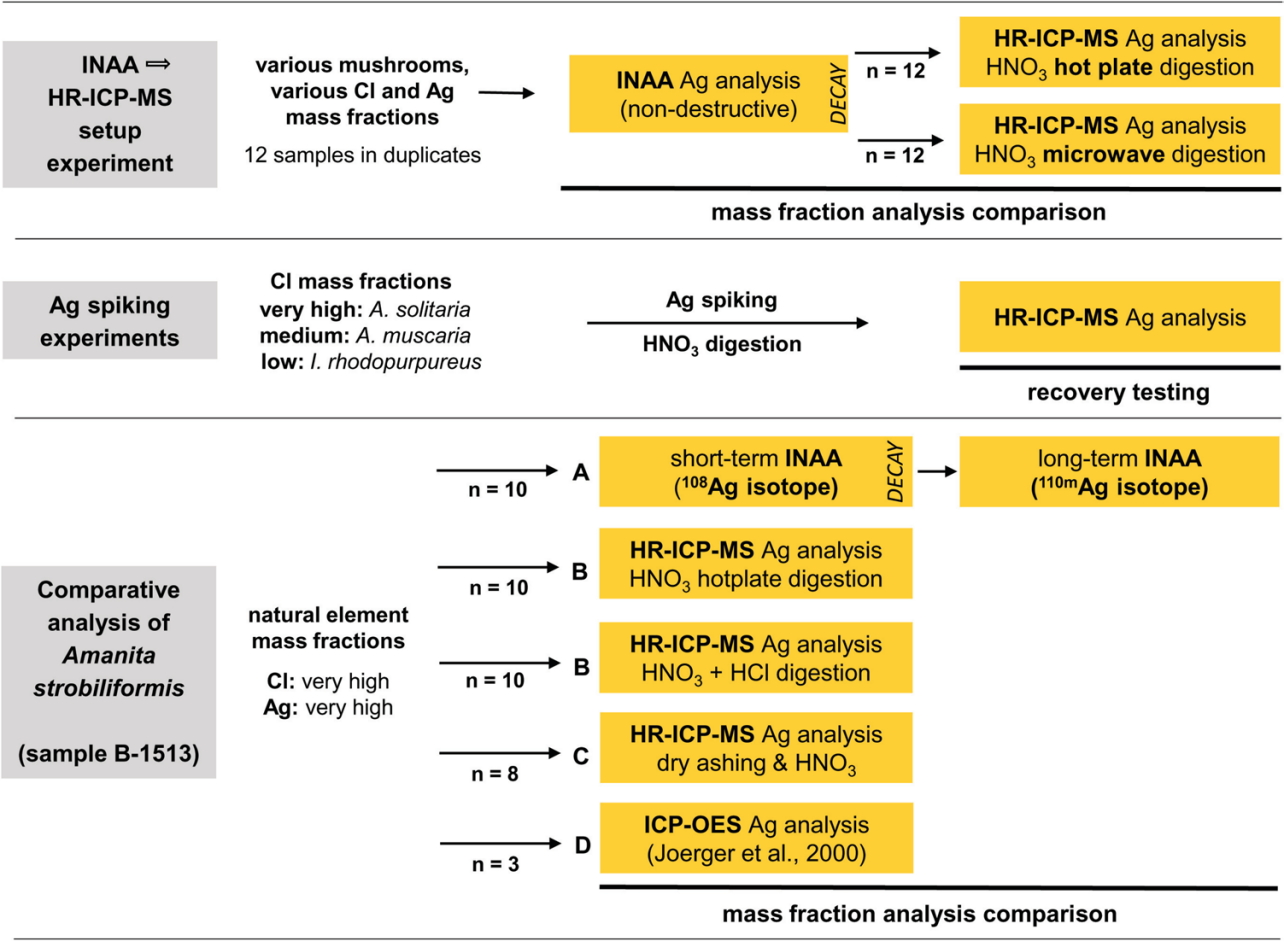
Flowchart depicting the analytical experiments conducted within this study

The analytical experiments performed in this study are depicted in a flowchart (Fig. 2).

### INAA → HR-ICP-MS Setup Experiment

For the comparison of Ag determination in mushroom samples by non-destructive INAA and destructive HR-ICP-MS analyses, we focused on mushroom samples with elevated Ag and both low and high Cl mass fractions. Based on the data we obtained in the past [21, 30 and unpublished results], we selected 12 mushroom samples (Table 1) from our archive (stored as dry mushroom powder in plastic vials). For additional information on the samples, see Supplementary Table S1, for how the mushroom samples were collected and processed see Borovička et al. [21].

Data for Cl mass fractions in the samples used in this study was collected within several sets of measurements before our experiments. Chlorine was determined by short-term INAA according to Řanda et al. [36]. Pelletized samples of circa 250 mg weight were transported by using a pneumatic system with a 3-s transport time to a nuclear reactor (LVR- 15, Nuclear Research Institute Řež, Czech Republic) [38]. The times of irradiation-decay-counting in mode 1-10-10 min have been used. Gamma-ray spectra of the irradiated samples, SRMs, and Cl standard (210 µg Cl) were measured using a coaxial high-purity germanium (HPGe) detector (PGT, USA) with relative efficiency of 25%, FWHM resolution 1.8 keV for the 1332.5 keV photons of ^60^Co; sample-to-detector distance of 5 cm was used. Chlorine was determined using the radionuclide ^38^Cl (half-life 37.3 min, gamma energies 1642.69 keV and 2167.68 keV). NIST SRM 1566b (Oyster Tissue) with certified Cl mass fraction of 5140 ± 100 mg kg^−1^ was used for quality control.

Silver was determined using short-term INAA with epithermal neutrons. This method allows sufficiently sensitive determination of Ag in mushroom samples, with irradiation resulting in relatively low-sample radioactivity. Mushroom powder of sample mass from 140 to 180 mg was pelletized using a hydraulic press (pellets 16 mm in diameter), weighed, and heat-sealed into nitric acid–cleaned polyethylene (PE) foil to obtain capsules 20 mm in diameter. For irradiation of these capsules, a Cd cylindrical box (diameter 25 mm, height 10 mm, wall thickness 1 mm) was used after cooling in liquid nitrogen. All samples were processed in duplicate. After a 30-s irradiation, gamma-ray spectra of the samples, SRM, and Ag standard (10 µg Ag) were measured for 1 min using the coaxial HPGe detector specified above. Depending on sample activity after irradiation, the count was done after a 30–120-s decay at a sample-to-detector distance of 2 cm for 1 min. Silver was determined using the radionuclide ^110^Ag (half-life 24.6 s, gamma energy 657.76 keV), the product of the neutron capture reaction on ^109^Ag [39]; epithermal activation is advantageous due to a high resonance integral of the ^109^Ag (n,γ) ^110^Ag reaction [40]. SRM 1566b with certified Ag mass fraction of 0.666 ± 0.009 mg kg^−1^ was used for quality control.

When activity of the samples reached the release limits, they could be safely processed for HR-ICP-MS analysis. Because we analyzed the samples in duplicates by INAA, the sample mineralization was conducted using (i) hotplate and (ii) microwave-assisted digestions.

For hotplate digestion, the pellets were unpacked from PE foils, placed in 60 mL perfluoroalkoxy alkane (PFA) digestion vessels (Savillex, USA), and weighed to correct the sample mass as traces of the mushroom pellets remained in the PE foil. Then, 6 mL HNO_3_ (double Teflon-distilled J.T. Baker, USA) were added, and the mixture was digested in tightly closed vessels on a hotplate overnight at 200 °C. The digests were then evaporated to a circa 1-mL drop, transferred to a volumetric flask, and diluted to 100 mL by 2% HNO_3_ in Millipore Milli-Q Element water (MQ water). Similarly, the samples were processed using the Microwave Accelerated Reaction System (MARS) 5 with XP 1500 Plus Teflon vessels (CEM Corporation, USA); 9 mL HNO_3_ were used, and the digestion was conducted at 200 °C (ramp time 15 min, hold time 15 min).

Silver concentrations in the resulting solutions were analyzed shortly after dilution in 2% HNO_3_ by HR-ICP-MS using the instrument Element 2 (Thermo Scientific, USA) housed at the Institute of Geology of the Czech Academy of Sciences, Prague. Standard analytical conditions of the instrument were utilized to analyze the solutions (Supplementary Table S2). Silver was measured in a low-resolution mode using ^107^Ag isotope and quantified via external calibration using blank and monoelement Ag solution (EPOND, Switzerland). Indium solution with concentration of 1 µg In L^−1^ was added via *Y*-piece in the sample introduction system as the internal standard. Typical sensitivity was circa 1500,000 cps/µg ^107^Ag L^−1^.

Mass fractions presented in this study are related to DM. The results of INAA and HR-ICP-MS are compared by using the analytical recovery (%R) according to the following relationship:

$$\%\text{Recovery}\hspace{0.17em}=\hspace{0.17em}[(\text{Ag})\text{HR}-\text{ICP}-\text{MS}/(\text{Ag})\text{INAA}]\hspace{0.17em}\times \hspace{0.17em}100\%$$

Correlation between variables were tested using the Spearman rank-correlation coefficient (*r*_*s*_).

### Ag-spiking Experiments

Two Cl-rich *Amanita* species were selected for the Ag-spiking sample experiment: *A. muscaria* and *A. solitaria* with 6200 mg Cl kg^−1^ and 21,000 mg Cl kg^−1^, respectively. Natural Ag mass fractions in these samples were determined by INAA according to Borovička et al. [21] as 4.92 and 11.5 mg kg^−1^, respectively. For comparison, *Imperator rhodopurpureus* sample with considerably lower Cl mass fraction of 860 mg kg^−1^ and natural Ag mass fraction of 2.33 mg kg^−1^ was used. For further details on the selected mushrooms, see Supplementary Table S1.

Monoelement Ag stock solution (1 g Ag L^−1^, Analytika, Czech Republic; nitrate form) and diluted solutions containing 20 mg Ag L^−1^, 5 mg Ag L^−1^, and 1 mg Ag L^−1^ were used to spike the powdered mushroom samples of 200 ± 10 mg weight to achieve “spiked Ag mass fractions” in the range of circa 10–1000 mg kg^−1^ in *A. muscaria*, 16–950 mg kg^−1^ in *A. solitaria*, and 7–1250 mg kg^−1^ in *I. rhodopurpureus*. Then, the samples were digested in 60 mL PFA vessels on the hotplate overnight and analyzed by HR-ICP-MS in the same way as described above. The analytical recovery (%R) was calculated according to the following relationship:

%Recovery = [(measured Ag mass fraction) HR-ICP-MS/(calculated Ag-spiked mass fraction) HR-ICP-MS] × 100%

### Comparative Analysis of *Amanitastrobiliformis*

In the last series of experiments, we selected *A. strobiliformis* sample B-1513 from our archive with a relatively high Ag mass fraction (expected to be above 700 mg kg^−1^) (Supplementary Table S1).

(A) Pelletized samples of 250 ± 10 mg DM were analyzed in ten replicates by the short-term INAA with thermal neutrons (without Cd boxes) according to Řanda et al. [36]. After a 60-s irradiation, gamma-ray spectra of the samples, SRM, and Ag standard (50 µg Ag) were measured using the coaxial HPGe detector specified above. The count was done after a 5-min decay at sample-to-detector distance of 11 cm for 5 min. Silver was determined using the radionuclide ^108^Ag (half-life 2.39 min, gamma energy 633 keV), the product of the neutron capture reaction on ^107^Ag [39].

After 2 weeks of decaying, the samples were reanalyzed using the long-term INAA according to Řanda and Kučera [41]. Briefly, after a 2-h irradiation, gamma-ray spectra of the samples, SRM, and Ag standard (25 µg Ag) were measured using a coaxial HPGe detector (Ortec, USA) with relative efficiency of 53%, FWHM resolution 1.76 keV for the 1332.5 keV photons of ^60^Co. The count was done after circa 1 month of decaying at a sample-to-detector distance of 10 cm for 2 h (mushrooms) or more (SRMs). Silver was determined using the radionuclide ^110 m^Ag (half-life 249.78 days, gamma energy 657.76 keV), the product of the neutron capture reaction on ^109^Ag [39]. NIST SRM 2781 (Domestic Sludge) with certified Ag mass fraction of 97.6 ± 6.5 mg kg^−1^ and SRM 1566b were used for quality control of both INAA variants.

Note: A minimum sample mass of 100 mg SRM 2781 should be used for analytical determinations to be related to the certified values provided. However, such sample mass could not be measured under the described conditions due to a high dead time of the detector. We therefore used a 50-mg sample mass in duplicate and report mean value of both measurements.

(B) Two other series of *A. strobiliformis* B-1513 samples (250 ± 10 mg DM) were analyzed by HR-ICP-MS. Ten replicates were digested on a hotplate with 6 mL HNO_3_ overnight as described above. Together, another ten replicates were digested in a mixture of 5 mL of HCl and 3.5 mL of HNO_3_ (J.T. Baker, USA, double Teflon-distilled in-house) according to Daskalakis et al. [42]. After digestion, the vessels were opened and the digests evaporated to a 1–2-mL drop which was transferred to plastic volumetric flask and diluted to 25 mL with MQ water. Then, the samples were 10 × diluted with 2% HNO_3_ and Ag was immediately measured by HR-ICP-MS as described above. SRM 1566b was used for the quality control of the HR-ICP-MS procedure in both digestion variants.

(C) Eight replicates of *A. strobiliformis* B-1513 were processed by the dry ashing method in a muffle furnace as used by Falandysz et al. [43–45]. The samples of 209 ± 3 mg DM were ashed in quartz glass crucibles initially at 200 °C (hold for 30 min), then at 250 °C (hold for 30 min), then at 300 °C (hold for 30 min), then at 350 °C (hold for 30 min), and finally at 420 °C for 8 h. The stepwise increase of temperature was 6 °C per min. The residue was wetted with concentrated HNO_3_ (< 0.1 mL) and re-ashed for about 1 h at 300 °C to whiten it. The resulting residue was dissolved in 5 mL of 10% HNO_3_, further diluted with 2% HNO_3_, and analyzed by HR-ICP-MS as described above.

(D) Last, three replicates of *A. strobiliformis* B-1513 were processed using a method adopted from Joerger et al. [46]. Samples of 251 ± 4 mg DM were digested using HNO_3_ on the hotplate overnight as described above. The resulting digests were reduced to circa 0.5 mL on the hotplate in open vessels, 3 mL of concentrated HCl were added, and closed vessels were further heated on the hotplate at 180 °C for 2.5 h. After that, the digests were diluted with freshly prepared solution of *aqua regia*, which consists of one-part *aqua regia* and three parts MQ water, to a final volume of 25 mL in glass volumetric flasks. Internal mushroom reference material M-122 (*Boletus reticulatus*) was processed in the same way for quality control, but sample mass of 526 mg was used, and the final solution was only diluted to 10 mL. Silver concentrations in the solutions were analyzed immediately after dilution by inductively coupled plasma optical emission spectroscopy (ICP-OES) using the instrument Agilent 5100 SVDV (Agilent, USA) housed at the Institute of Geology of the Czech Academy of Sciences, Prague (for instrumental parameters see Supplementary Table S2). Silver was quantified via external calibration using blank and monoelement Ag solution (ASTASOL, Analytika, Czech Republic); the wavelength of the emission peak used for Ag determination was 328.068 nm.

### SEM and XRD Analysis of the Precipitate

In many digested mushroom samples with both naturally and artificially high Ag mass fractions, one or more fine gray-white grains could be observed in the PFA vessels. We therefore selected a sample of *A. strobiliformis* (B-194b) containing 993 mg Ag kg^−1^ [30] and used it for a detailed analysis of this precipitate. A portion of 508 mg dry biomass was digested on a hotplate with 11 mL of HNO_3_ overnight. The formed precipitate was then carefully washed in the PFA vessel by water and acetone, dried, and weighed, yielding total mass of 0.64 mg. This precipitate was then investigated by SEM (TESCAN VEGA3 XMU, Czech Republic) and XRD using the Bruker D8 DISCOVER instrument (Bruker, USA).

## Results

### INAA → HR-ICP-MS Setup Experiment

The comparison between Ag determination by INAA and HR-ICP-MS is presented in Table 1. The Ag concentration values found for SRM 1566b are in accordance with the certified range for both the INAA and HR-ICP-MS determinations. However, in mushroom samples, the comparison between INAA and HR-ICP-MS shows a significant discrepancy. In the case of microwave digestion, only three out of 12 samples showed a good match (± 10%) (Table 1). A good match was particularly observed in samples with both low Ag and Cl contents (*Boletus reticulatus*) or with high Ag but low Cl (*Lycoperdon excipuliforme*). On the other hand, samples with high Cl and Ag contents (*Amanita* species in particular) showed the lowest agreement. In the hotplate digestion, the agreement between INAA and HR-ICP-MS was better, but recovery was still below 90% for six out of 12 samples. In both hotplate and microwave digestion methods, there was a significant negative correlation between the Cl mass fraction and the recovery (*r*_*s*_ = − 0.87, 0.01 level, and *r*_*s*_ = − 0.59, 0.05 level, respectively).

**Table 1:** Determination of Ag mass fractions in mushroom sample with various Cl contents using short-term INAA and HR-ICP-MS. INAA results for Ag are presented with statistical uncertainty (concentration error) of determination. Analytical recovery^a^ values lower than 90% are indicated in bold

Mushroom species	Sample ID	Cl (INAA) [mg kg^−1^]^b^	Ag (INAA) [mg kg^−1^]	ICP-MS: microwave digest		Ag (INAA) [mg kg^−1^]	ICP-MS: hotplate digest	
				Ag [mg kg^−1^]	Ag (%)R^a^		Ag [mg kg^−1^]	Ag (%)R^a^
*Lycoperdon excipuliforme*	AGP-3	58.3	63.1 ± 0.2	63.8	101	62.6 ± 0.4	59.3	95
*Paxillus involutus*	B-58	268	48.0 ± 0.3	44.3	92	48.6 ± 0.3	45.4	94
*Imleria badia*	AGP-7	1170	23.8 ± 0.2	21.1	**89**	23.7 ± 0.2	23.9	101
*Amanita crocea*	AGP-19	2320	319 ± 2	144	**45**	329 ± 2	237	**72**
*Boletus reticulatus*	M-122	2880	7.25 ± 0.1	7.27	100	7.20 ± 0.1	6.99	97
*Agaricus bernardii*	B-731	5000	26.1 ± 0.2	17.6	**67**	26.3 ± 0.2	26.2	100
*Cortinarius cinnamomeus*	B-611	5490	289 ± 2	52.4	**18**	292 ± 2	140	**48**
*Amanita muscaria*	B-16	10,500	47.5 ± 0.3	16.7	**35**	47.6 ± 0.3	48.3	101
*Amanita strobiliformis*	B-188c	14,100	850 ± 5	19.6	**2**	852 ± 5	82.5	**10**
*Amanita strobiliformis*	B-212b	15,000	257 ± 2	18.4	**7**	257 ± 2	79.9	**31**
*Amanita strobiliformis*	B-194a	26,100	672 ± 6	19.1	**3**	598 ± 7	189	**32**
*Amanita strobiliformis*	B-196	27,800	211 ± 2	26.3	**13**	213 ± 2	91.0	**43**
NIST SRM 1566b^c^	-	5140 ± 100^d^	0.65 ± 0.02	0.66	101	0.71 ± 0.03	0.66	93

^a^Analytical recovery: %Recovery = [(Ag)ICP-MS/(Ag)INAA] × 100%

^b^Statistical uncertainty (concentration error) for Cl determination was circa 1% (rel.) in most samples

^c^Ag certified mass fraction for SRM 1566b is 0.666 ± 0.009 mg kg^−1^

^d^Certified mass fraction provided by NIST is indicated

### Ag-spiking Experiments

**Fig. 3:**
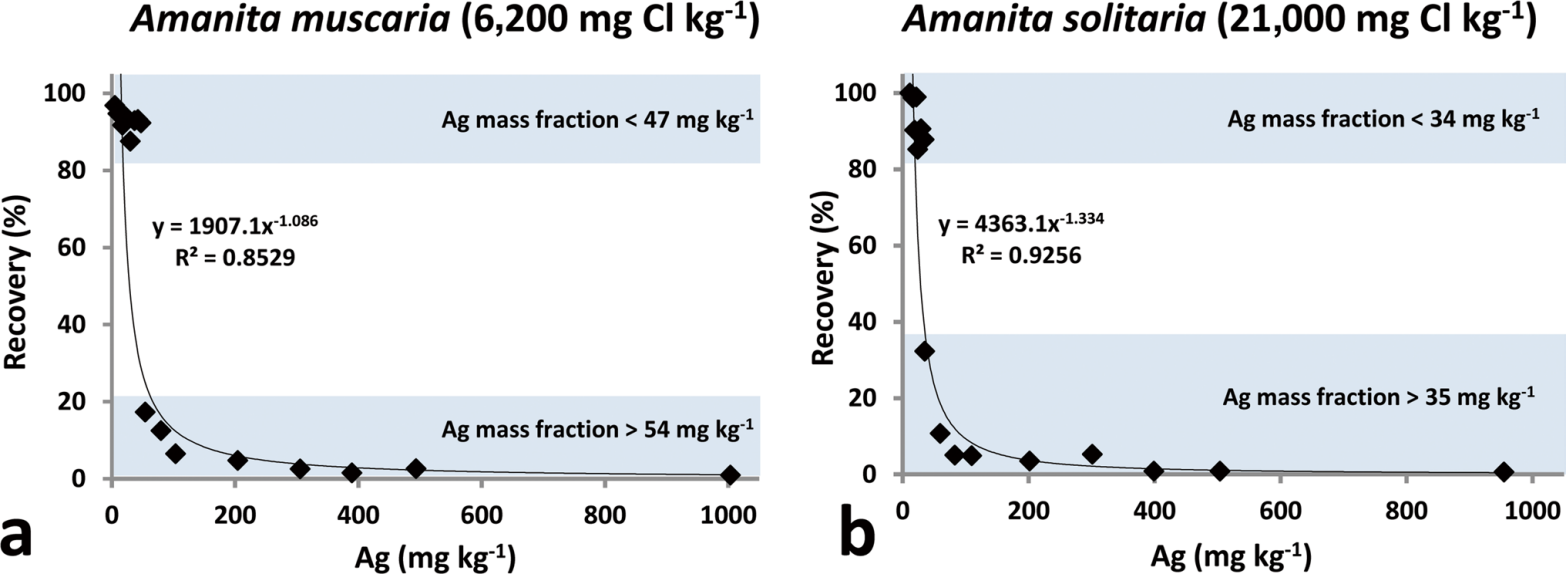
Analytical recoveries (%) for Ag-spiked *Amanita* samples. **a** *A. muscaria*. **b** *A. solitaria*

**Fig. 4:**
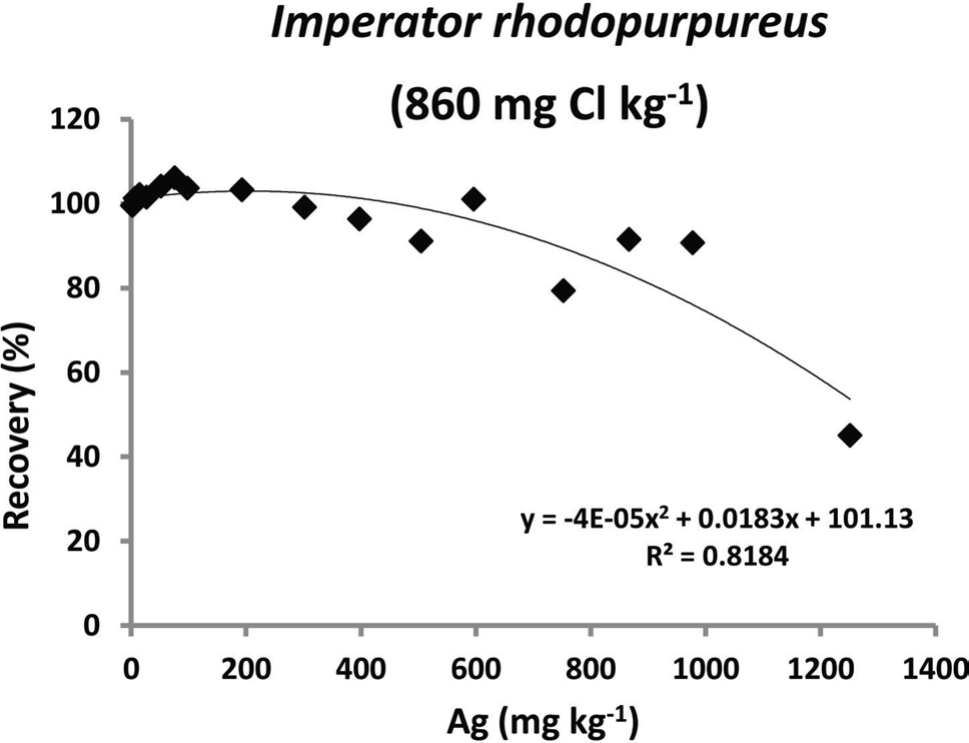
Analytical recoveries (%) for Ag-spiked *Imperator rhodopurpureus* sample

In the Cl-rich *Amanita* samples, the analytical recovery decreases dramatically with the amount of Ag added. The recoveries were somewhat better in *A. muscaria*, where Ag mass fractions up to 47 mg kg^−1^ were more or less satisfactorily determined (Fig. 3a, recovery 88–97%). In *A. solitaria* (with circa 3.5 × higher Cl mass fraction), Ag mass fractions over 35 mg kg^−1^ could not be accurately determined (Fig. 3b, recovery 32% and lower). In *I. rhodopurpureus* with a relatively low Cl mass fraction, most of the recoveries were within a 90–110% range, with only the highest Ag spike (expected Ag mass fraction of 1250 mg kg^−1^) yielding low recovery of 45% (Fig. 4). See also Supplementary Table S3.

### Comparative Analysis of *Amanitastrobiliformis*

**Fig. 5:**
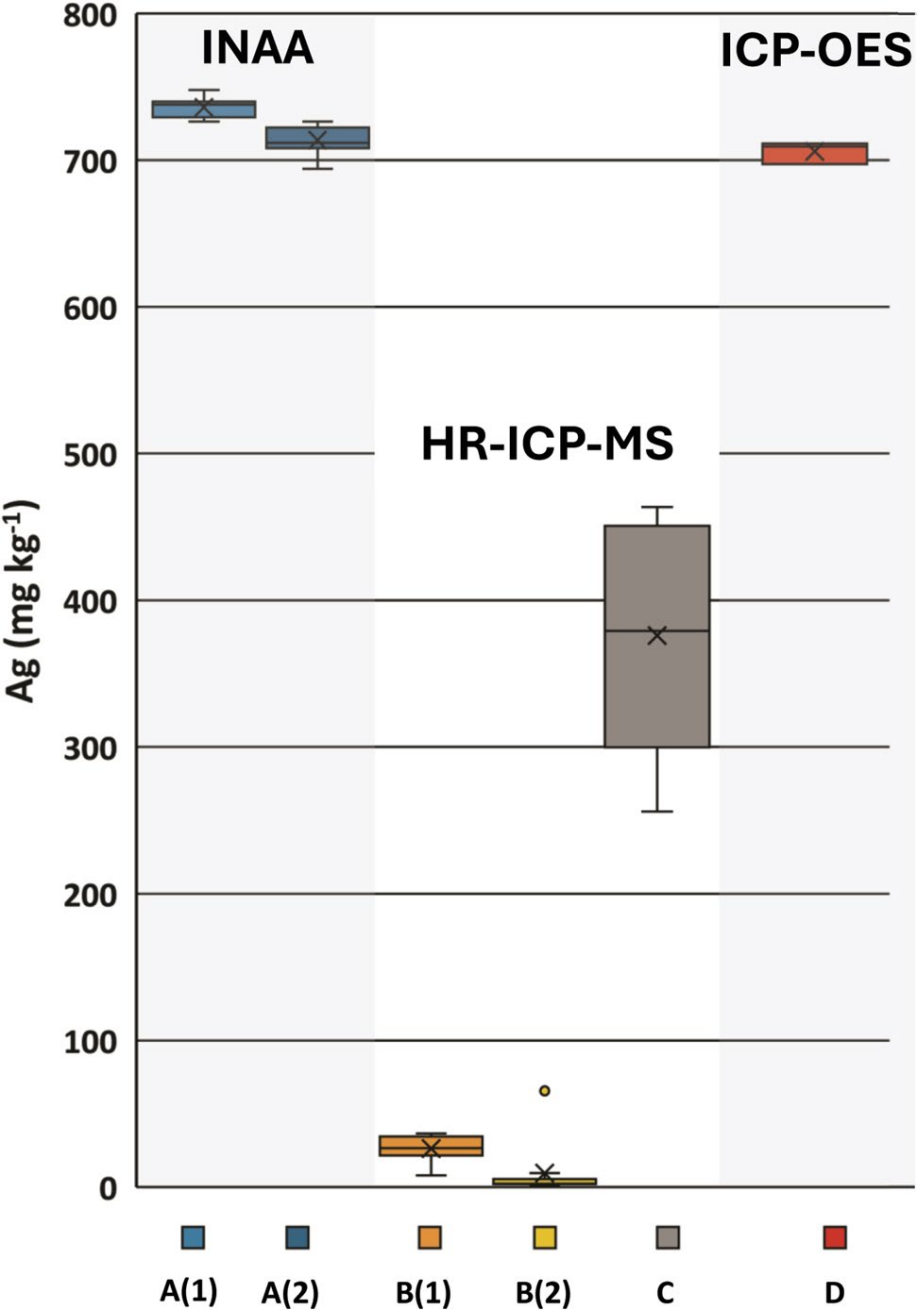
Box plots showing the results of Ag analysis in *Amanita strobiliformis* B-1513. A(1): non-destructive short-term INAA (^108^Ag); *n* = 10. A(2): non-destructive long-term INAA (^110 m^Ag); *n* = 10. B(1): HR-ICP-MS after HNO_3_ digestion; *n* = 10. B(2): HR-ICP-MS after HCl–HNO_3_ digestion according to Daskalakis et al. [42]; *n* = 10. C: HR-ICP-MS after dry ashing and HNO_3_ dilution; *n* = 8. D: ICP-OES after two-step acid digestion according to Joerger et al. [46]; *n* = 3

(A) According to the short-term INAA utilizing the ^108^Ag isotope, the mean Ag mass fraction (± SD) in *A. strobiliformis* (*n* = 10) was 736 ± 6 mg kg^−1^ (Fig. 5). Silver mass fraction in SRM 1566b could not be determined due to a high detection limit under these analytical conditions (approx. 2 mg kg^−1^). However, the measured Ag mass fraction of 99.9 ± 7.8 mg kg^−1^ in the SRM 2781 (the mean is presented with statistical uncertainty) fits the certified range. The mean Ag mass fraction (± SD) obtained for the same dataset by long-term INAA utilizing the ^110 m^Ag isotope was somewhat lower: 714 ± 9 mg kg^−1^ (Fig. 5), but the results obtained by this method for Ag in SRM 1566b (0.64 ± 0.01 mg kg^−1^; subtly below the certified range) and SRM 2781 (92.0 ± 0.93 mg kg^−1^; matches the certified range) were highly satisfactory; results for both SRMs are presented with statistical uncertainty. On average, the Ag mass fractions determined in *A. strobiliformis* by long-term INAA (^110 m^Ag) were 3% lower than those determined by the short-term INAA (^108^Ag).

(B) In samples digested with HNO_3_, mean Ag mass fraction (± SD) measured by HR-ICP-MS was 26.2 ± 8 mg kg^−1^, whereas in samples digested in HCl–HNO_3_ mixture even lower: 9.42 ± 19 mg kg^−1^ (median 2.29 mg kg^−1^). The measured Ag mass fractions in SRM 1566b were nearly identical for both digestion procedures: 0.67 and 0.66 mg kg^−1^, which fit the certified range. It should be noted that the hotplate digestion with a HCl–HNO_3_ mixture did not work well and yielded undigested sticky organic residues adhered to the bottom of the PFA vessels. However, the significantly lower results obtained for Ag in the samples digested by the HCl–HNO_3_ mixture cannot be attributed to an insufficient digestion of the biomass as the calculated mass fractions of other selected elements in this sample set were more or less identical (± 5%; Supplementary Table S4) when compared to the HNO_3_ digestion. A precipitate in the form of one or few tiny gray-white grains was observed in all vessels after the HNO_3_ digestion but not after digestion with HCl–HNO_3_.

(C) Silver mass fractions measured by HR-ICP-MS after dry ashing were much higher than those obtained after the HNO_3_ digestion (376 ± 72 mg kg^−1^) but still about half of those obtained by INAA (for data see Supplementary Table S5).

(D) Yielding an average Ag mass fraction of 706 ± 6 mg kg^−1^ (Supplementary Table S5), the procedure modified from Joerger et al. [46] appeared to be well comparable with the long-term INAA (714 ± 9 mg Ag kg^−1^); the Ag hyperaccumulation ability of *A. strobiliformis* has thus been finally confirmed by other method than INAA. Silver mass fraction obtained for the internal mushroom reference material M-122 was 7.0 mg kg^−1^ which well corresponds to our internal reference value of 7.3 mg kg^−1^ (see also Table 1).

### Investigation of the Precipitate

**Fig. 6:**
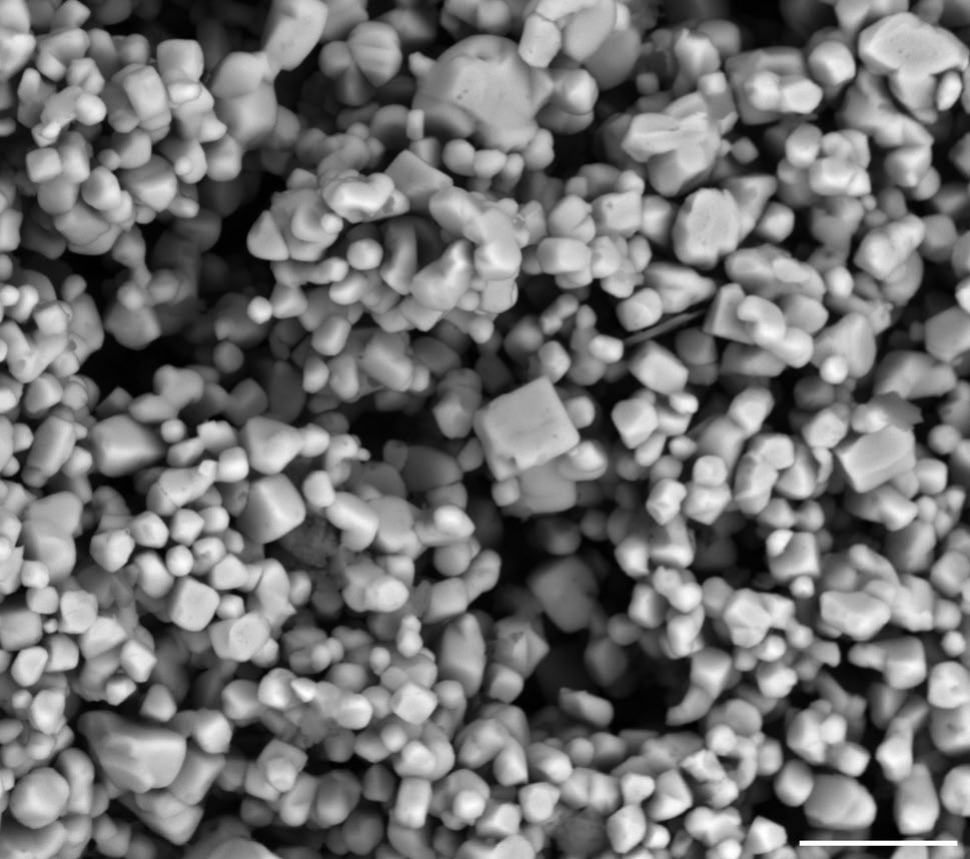
Scanning electron microscopy image of AgCl precipitate formed during the hotplate HNO_3_ digestion of Ag-hyperaccumulating mushroom *Amanita strobiliformis *B-194b. Scale bar = 5 µm

**Fig. 7:**
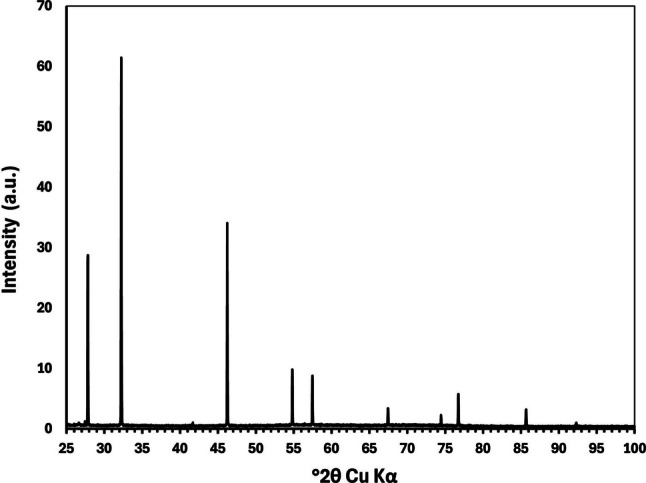
X-ray diffraction pattern of the precipitate, corresponding to AgCl, formed during the hotplate HNO_3_ digestion of Ag-hyperaccumulating mushroom *Amanita strobiliformis*

The SEM micrograph of the precipitate formed during the digestion of *A. strobiliformis* sample B-194b is presented in Fig. 6. The precipitate consisted of a mass of tiny cubic crystals which, as we repeatedly tested, were easily soluble in concentrated ammonia. The XRD analysis (Fig. 7) unambiguously determined these crystals as silver chloride (Crystal Open Database, entry number 9011666), which is consistent with our initial hypothesis and also all the characteristics observed.

## Discussion

Digestion of biogenic materials in hot concentrated HNO_3_ is a common procedure for assessing their elemental content by instrumental techniques [47–50]. But as suggested by our study of mushrooms, Ag determination may pose a serious problem for all analytical methods using this digestion procedure in samples rich in both Ag and Cl. Precipitation of AgCl is a rapid process and well-known phenomenon [51]. Several studies have already addressed the subject of low Ag recovery after HNO_3_ digestion in seafood [42, 52, 53], NIST SRM 1597 (Non-Fat Milk Powder) [54], microbial biomass [46], rat urine [55], water samples [56], copper shales [57], and soils/sediments/sludges [58, 59]. The particular novelty of our work lies in the finding that in mushrooms the Ag values detected by the HR-ICP-MS analysis after the HNO_3_ digestion may be indeed dramatically lower (< 90%), making it virtually impossible to correctly identify the Ag-hyperaccumulating mushroom species.

Our data (Table 1) indicates that low Ag recovery after HNO_3_ digestion particularly appears in samples with high both Ag (tens of mg kg^−1^ or higher) and Cl (circa 5000 mg kg^−1^ or higher) in the matrix. Such a situation, however, may possibly include not only mushrooms and other biological tissues, but also environmental samples such as Ag-contaminated organic soils or sewage sludge [59–63]. The high Ag mass fractions determined in our mushroom samples by INAA are obviously real: this has not only been confirmed by the analysis of Ag in two SRMs, but especially by the analytically independent routes for Ag determination by INAA, the so-called self-validation principle [64], in this particular case represented by the use of ^110^Ag, ^108^Ag, and ^110 m^Ag isotopes.

It is worth noting that the difference between microwave and hotplate digestion procedures (Table 1), where better Ag recoveries, were obtained in the latter method. The same phenomenon was observed by Daskalakis et al. [42] who claim that prolonged contact of the acid mixture with the tissue on the hotplate may aid in the dissolution of insoluble phases. Crecelius and Daskalakis [53] and Daskalakis et al. [42] speculated that low Ag recoveries in their marine samples may not only be attributed to high Cl but also to a possible presence of a HNO_3_-insoluble Ag form in the samples. In this study, however, we have shown that the low Ag recoveries in mushrooms after acid digestion must be attributed to the precipitation of AgCl which has been unambiguously confirmed in the digest, probably for the first time in a natural sample.

This study further demonstrates that the analysis of SRMs alone does not necessarily provide confidence in the accuracy of the results of the analysis of unknown samples. We came across the whole problem essentially by chance, as our default method was INAA, where the AgCl precipitation process does not occur due to its non-destructive character. Moreover, our knowledge of the high Cl mass fractions in *A. strobiliformis*, also detected by INAA, contributed to the hypothesis and problem solution.

High Ag mass fractions in mushrooms were first pointed out by Byrne et al. [6], who also used INAA. Later, Borovička et al. [21] demonstrated that Ag accumulation in mushrooms is a common phenomenon in both clean and polluted environments. In Table 2, we present studies reporting Ag mass fractions in mushrooms. The nitric acid digestion (and its variants additionally using, e.g., hydrogen peroxide) appears to be the most common current method of the Ag analysis. However, it is not reasonable to assume that a large proportion of the published results are wrong. Our spiking experiments have indicated that in mushroom samples with elevated (6200 mg kg^−1^) or highly elevated (21,000 mg kg^−1^) Cl mass fractions, Ag recoveries are unacceptably low at levels higher than approx. 50 and 30 mg kg^−1^, respectively (Fig. 3). The problem of erroneous Ag determination may therefore arise especially in *Amanita* species (e.g., 12) or other (including the not yet known) Ag and Cl accumulators from either pristine or Ag-polluted environments.

**Table 2:** Studies reporting Ag mass fractions in mushrooms

Digestion reagent(s)	Instrumental analysis	Reference
HNO_3_	ICP-MS	[23, 24, 65–70]
HNO_3_	HR-ICP-MS	[71–73]
HNO_3_	GFAAS	[9, 74, 75]
HNO_3_	ICP-OES	[12, 76–83]
HNO_3_	ICP-QQQ-MS	[26, 84]
HNO_3_-H_2_O_2_	ICP-MS	[85]
HNO_3_-H_2_O_2_	FAAS	[86]
HNO_3_-H_2_O_2_	GFAAS	[87–91]
HNO_3_-H_2_SO_4_	GFAAS	[92]
HNO_3_-H_2_SO_4_-H_2_O_2_	AAS	[93]
Dry ashing, HNO_3_	ICP-MS	[94]
Dry ashing, HNO_3_	FAAS	[43–45]
(Non-destructive)	INAA	[5, 6, 21, 26, 30, 36, 74, 95–98]

Crecelius and Daskalakis [53] and Daskalakis et al. [42] suggested adding HCl to HNO_3_ in the digestion step to achieve values comparable to the good results of ultrasonic slurry sampling–graphite furnace atomic absorption spectrometry (USS–GFAAS) and INAA. According to the authors, due to the considerable enrichment of the solution with Cl ions, the formation of soluble AgCl_*n*_^1–*n*^ complexes would promote the dissolution of Ag and its stabilization in solution. But in the case of *A. strobiliformis* with Ag content above 700 mg kg^−1^, this procedure proved to be inappropriate, because the addition of HCl led to an even lower Ag recoveries than in the case of using HNO_3_ alone (Fig. 5). Furthermore, the digestion procedure did not work well and yielded undigested organic residues which was also observed for a*qua regia* digestion of microbial cells by Joerger et al. [46]. The ashing procedure was also not helpful to reach accurate results for Ag but led to much better yields than the one-step acid digestion procedures.

The phenomenon of Ag hyperaccumulation in mushrooms deserves detailed research, both for its biological significance and possible biotechnological applications in the future. Many *Amanita* species occur in North America [99], the diversity of the European taxa is much higher than previously believed [100] and many of them likely hyperaccumulate Ag. However, research on these mushrooms may end before it begins unless a suitable analytical method capable of detecting high Ag mass fractions in mushroom biomass is chosen. In this sense, INAA seems ideal, but it is becoming less and less available due to the cancellation of research reactors around the world.

Therefore, the two-step digestion method proposed by Joerger et al. [46] is a good alternative for accurate Ag determination in *Amanita* spp. and other Ag-hyperaccumulating mushrooms. However, it is necessary to ensure (i) complete dissolution of the precipitated AgCl after the HNO_3_ digestion step [101] and (ii) the subsequent stability of the diluted solution because of the risk of AgCl precipitation before and during the measurement by ICP-based techniques.

Finally, the precipitation of AgCl during sample digestion must also be considered when studying the isotopic composition of Ag in mushrooms, since this process results in the isotopic fractionation of Ag [102].

## Conclusion

Determination of Ag mass fractions in mushrooms using acid digestion procedures may be problematic in mushrooms with high Ag contents, i.e., from polluted sites or in Ag (hyper)accumulators. This is because many mushrooms contain high levels of Cl, which leads to precipitation of insoluble AgCl during sample digestion and consequently to incorrect determination of Ag (values up to 98% lower than in reality). Some of the Ag mass fraction data for mushrooms published in the past are possibly affected by this phenomenon. Unfortunately, this problem cannot be detected by the use of certified reference materials during the analysis. In this context, INAA appears to be the most suitable method for the determination of Ag in mushrooms. Alternatively, a method proposed by Joerger et al. [46] was proved to work well for Ag determination in a Cl-rich *Amanita* sample by ICP-OES. In any case, when measuring Ag in materials of unknown composition after acid digestion, care must always be taken to consider the possible influence of the Cl content of the sample itself.

## Supplementary Information

Below is the link to the electronic supplementary material.

Supplementary file1 (XLSX 31.6 KB)

## Data Availability

No datasets were generated or analysed during the current study.
